# Wheat Germ Oil and Propolis Decrease Parasite Burden and Restore Marked Histopathological Changes in Liver and Lung in Mice with Chronic Toxoplasmosis

**DOI:** 10.3390/ani12223069

**Published:** 2022-11-08

**Authors:** Ashraf Mohamed Barakat, Hassan Ali Mohamed El Fadaly, Ahmed Gareh, Khaled A. Abd El-Razik, Fatma Abo Zakaib Ali, Amira A. Saleh, Sabry A. S. Sadek, Naief Dahran, Abd El-Nasser G. El-Gendy, Manal F. El-Khadragy, Ehab Kotb Elmahallawy

**Affiliations:** 1 Department of Zoonotic Diseases, National Research Centre, El Buhouth St., Dokki, Giza 12622, Egypt; 2 Department of Parasitology, Faculty of Veterinary Medicine, Aswan University, Aswan 24101, Egypt; 3 Department of Animal Reproduction, Veterinary Research Institute, National Research Centre, Dokki, Giza 12622, Egypt; 4 Department of Pathology and Clinical Pathology, Faculty of Veterinary Medicine, Sohag University, Sohag 82524, Egypt; 5 Department of Human Medical Parasitology, Faculty of Medicine, Zagazig University, Zagazig 44519, Egypt; 6 Department of Anatomy, Faculty of Medicine, University of Jeddah, Jeddah 21959, Saudi Arabia; 7 Medicinal and Aromatic Plants Research Department, Pharmaceutical and Drug Industries Research Institute, National Research Centre, Dokki, Giza 12622, Egypt; 8 Department of biology, College of Science, Princess Nourah bint Abdulrahman University, P.O. Box 84428, Riyadh 11671, Saudi Arabia; 9 Department of Zoonoses, Faculty of Veterinary Medicine, Sohag University, Sohag 82524, Egypt

**Keywords:** wheat germ, propolis, parasite burden, histopathological changes, liver, lung, chronic toxoplasmosis, mice

## Abstract

**Simple Summary:**

Toxoplasmosis is a major parasitic zoonotic disease with worldwide distribution. It has a wide range of reservoirs and can result in abortion and permanent congenital anomalies in fetuses. Several studies have reported an alarming increase in parasite resistance to available drugs, making treatment of infection a major challenge, especially in the chronic stage of the disease. The present study investigated the antiparasitic effect of wheat germ oil and propolis on the chronic stage of *Toxoplasma gondii* in experimentally infected mice. The parasitological load in the liver and lungs of treated animals was quantified and compared with positive control animals by parasitological methods and molecular techniques. Histopathological changes as a result of infection were also investigated. The results demonstrate promising ameliorative effects of the combination of wheat germ oil and propolis against parasite burden and restoration of histopathological lesions in the liver and lungs during chronic toxoplasmosis infection.

**Abstract:**

Toxoplasmosis is a parasitic zoonotic disease with a worldwide distribution. Its effects can be critical in immunocompromised patients. However, there is a limited availability of effective, low-toxicity drugs against this disease, particularly in its chronic form. The present study evaluated the effect of propolis and wheat germ oil (WGO) as safe, natural products to reduce *Toxoplasma* cysts in experimentally infected mice. For the experiment, five groups (10 mice per group) were examined: Group 1: negative control (noninfected, nontreated); Group 2: positive control (infected, nontreated); Group 3: infected and treated with WGO at a dose of 0.2 mg/1.5 mL per kg body weight/day; Group 4: infected and treated with 0.1 mL propolis extract/day; and Group 5: infected and treated with a combination of WGO and propolis at the same doses as Group 3 and 4. After the mice were sacrificed, liver and lung specimens underwent histopathological examination, and the parasite burden was investigated by parasitological methods and quantified using real-time polymerase chain reaction. Notably, the results showed a substantial decrease in parasitic burden in Group 5 compared to the control group. These results were further confirmed by molecular analysis and quantification of the DNA concentration of the *Toxoplasma* P29 gene after treatment in all tested samples. Furthermore, the combination of propolis and WGO restored all histopathological changes in the liver and lungs. Taken together, these findings provide remarkably promising evidence of the effects of the combination of WGO and propolis against chronic toxoplasmosis in mice.

## 1. Introduction

*Toxoplasma gondii* is one of the most common coccidian parasites of man and other animals and is considered an important zoonotic parasite with a worldwide distribution [1]. This parasite infects herbivorous, omnivorous, and carnivorous animals, and approximately one-third of humanity has been exposed to it [2,3]. *T. gondii* is a heterogeneous parasite that requires both final (feline) and intermediate (any warm-blooded animal or human) hosts to complete its sexual and asexual life cycles [4]. The asexual phase of development takes place in different tissues of herbivorous or omnivorous intermediate hosts, whereas the sexual phase occurs in the intestine of definitive hosts [5]. Definitive hosts are members of the family Felidae, such as domestic cats [6]. Three infective stages of *T. gondii* are known, namely tachyzoites, bradyzoites (in tissue cysts), and sporozoites. Tachyzoites are typically crescent-shaped and invade host cells, where they proliferate asexually by repetitive division, resulting in tissue cysts that persist intracellularly [7]. Each tissue cyst has a thin, flexible wall that encircles hundreds of small, crescent-shaped bradyzoites. After the digestion of tissue cysts by cats, the wall is dissolved by proteolytic enzymes, and the free bradyzoites enter the epithelial cells of the small intestine, where they develop into schizonts. Oocysts of *T. gondii* are formed only in felids after consumption of any of the infective stages of *T. gondii,* and sporulation occurs outside of the cat within 1–5 days, dependent on airing and temperature [1]. Hosts may acquire *T. gondii* infection horizontally via oral ingestion of infectious oocysts from the environment and consumption of tissue cysts in undercooked meat or vertically by transplacental transmission of tachyzoites, spread via milk from mother to offspring or congenitally [8,9]. Recent studies have reported that water contaminated with oocysts is another probable source of Toxoplasma infection in humans and animals [10,11]. However, transmission by organ transplantation and blood transfusion is less common [12]. While *T. gondii* infection in immunocompetent humans is typically asymptomatic, there may be mild symptoms, including lymphadenopathy associated with fever, fatigue, muscle pain, sore throat, and headache [13]. Severe manifestations rarely occur in immunocompetent humans but include encephalitis, sepsis disorder, shock, myocarditis, and hepatitis [14]. There are also reports of spontaneous abortion or congenital defects if contracted for the first time during pregnancy [15]. Immunocompromised patients severely affected with *T. gondii* may experience reactivation of latent infection, leading to symptomatic disease [16,17,18]. Toxoplasmic encephalitis is a mutual sequela among AIDS patients infected with toxoplasmosis and is usually the result of the reactivation of latent tissue cysts [2]. *T. gondii* is capable of initiating severe disease in animals other than humans [1]. In the definitive host, cats infected with *T. gondii* appear depressed and anorexic and may die abruptly without noticeable clinical signs. Toxoplasmosis in sheep and goats can lead to great harm via embryonic loss and resorption, fetal death and mummification, abortion, stillbirth, and neonatal death. As the clinical signs of toxoplasmosis are non-specific and insufficiently distinctive, *T. gondii* infection must be diagnosed by biological, serological, histological, or molecular methods. Molecular methods, such as using polymerase chain reaction (PCR) to detect *T. gondii* DNA, have proved to be simple, sensitive, reproducible, and cost-effective and have been applied in diverse clinical trials, including on animals and humans [19]. The number of tachyzoites and proportional parasite burden is much higher during *Toxoplasma* infection in peripheral organs, such as the lung parenchyma and liver parenchyma, compared with parenchymal organs [20]. The most common treatments for acute toxoplasmosis are sulfadiazine and pyrimethamine (Daraprim). However, these drugs are believed to have little effect on subclinical infection. Difficult cases may be treated with diaminodiphenylsulfone, atovaquone, spiramycin, and clindamycin [21]. However, there are currently no available therapies to eliminate cysts that develop in the central nervous system of immunocompromised patients infected with chronic toxoplasmosis [22]. Natural medications extracted from plants, microbes, and animals play a substantial role in traditional medicine and provide new organic compounds for drug discovery research [23]. Thus, such compounds may provide new antiprotozoal drugs with high efficacy and low toxicity, which are urgently needed [24]. Wheat germ and propolis have been previously used as natural products to treat protozoal diseases [25,26]. Wheat germ (*Triticum aestivum* or Ganin Habit Al-Kamh), and its active component, lectin, are concentrated sources of vitamin E, folic acid, phosphorus, thiamin, zinc, magnesium, and essential fatty acids [27]. Clinically, wheat germ is beneficial for the treatment of *Giardiasis, Acanthamoeba, Trichomonas Vaginalis,* and the acute and chronic stages of infection with *Entamoeba histolyica* [24,26,28]. Wheat germ has also been shown to reduce the excretion of oocysts when used experimentally in immunosuppressed mice infected with Cryptosporidium oocysts [29]. Furthermore, combined therapy of nitazoxanide (NTZ) and wheat germ agglutinin (WGA) has shown therapeutic value against cryptosporidial infection [29]. Propolis, also known as bee gum, is created by honeybees from plant liquids as resins and adhesive exudates on leaf buds and plant wounds. Propolis has a long history of use as a natural medication for various disorders [30]. The essential components of propolis are phenolics, flavonoids, aromatic acids, caffeic acid and its esters, and cinnamic acids [31,32]. In in vitro studies, propolis extracts have shown antiparasitic activity against *T. gondii*, *T. vaginalis*, *Cryptosporidium, Leishmania donovani*, *Plasmodium falciparum*, and *Crithidia fasciculata,* and *Trypanosoma cruzi* [33,34,35,36,37,38]. However, few studies have investigated the potential effects of wheat germ oil (WGO) and propolis on *Toxoplasma* infection. Given prior findings of the value of WGO and propolis as antiprotozoal treatments, the present study evaluated the effect of combined WGO and propolis on mice experimentally infected with *Toxoplasma,* including effects on parasite burden and histopathological changes in the liver and lung during chronic toxoplasmosis.

## 2. Materials and Methods

### 2.1. Ethical Considerations

This study was approved by the research ethics committee of the Veterinary Research Institute, National Research Centre, Egypt (institutional approval board number 19/139/2020).

### 2.2. Materials

WGO and propolis were obtained from local Egyptian markets selling medicinal plants and herbs. Characterization of silylated primary metabolites and metabolite identification, including WGO and propolis, was performed using gas chromatography–mass spectrometry GC-MS analysis [39,40,41], as performed in a previous study (unpublished data). Tachyzoites of *Toxoplasma gondii* (ME49 or Avirulent strain) were provided by the Department of Zoonoses, Veterinary Research Institute, National Research Center, Egypt. Mice underwent intraperitoneal inoculation with parasite materials. Tachyzoites were collected from the peritoneal cavity of infected mice 72 h after inoculation. The number of parasites was calculated using a hemocytometer, and the number was adjusted to 10^3^/mL by attenuation in the applicable volume of saline [42].

### 2.3. Experimental Procedure

**Figure 1:**
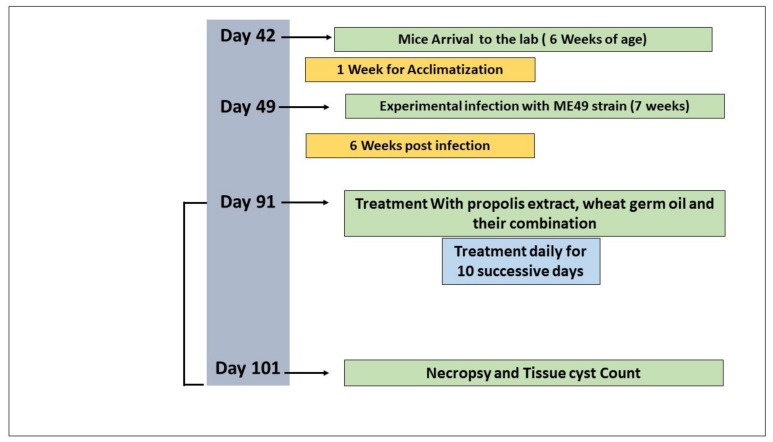
Experimental protocol of the present study.

The overall design of the experiment is shown in Figure 1. A total of 50 6-week-old female Swiss albino laboratory mice (weighing 25–30 g) were acclimatized for one week before the experiment. Animals were kept in well-ventilated cages with ad libitum access to a standard diet and water [43]. The mice were experimentally infected by repeated intraperitoneal injection of mice every 8 weeks with the ME49 parasite strain (Avirulent strain) using 0.1 mL of brain suspension containing 10^3^ cysts of previously infected mice [25]. Six weeks post-infection, herbal ingredients were orally administered to mice daily using a stomach tube for 10 days [44]. The mice were divided into the following five groups (10 mice per group): Group 1 (G1), negative control: noninfected, nontreated mice; Group 2 (G2), positive control: infected, nontreated mice; Group 3 (G3): infected and treated with 0.1 mL propolis extract/day [45]; Group 4 (G4): infected and treated with WGO at a dose of 0.2 mg/1.5 mL/per kg body weight/day [46]; and Group 5 (G5): infected and treated with a combination of WGO and propolis using the same dose in G3 and G4. Mice were assessed daily for any clinical signs.

### 2.4. Parasitological Assessment

Mice from all groups were sacrificed, and their livers and lungs were dissected and examined for gross lesions. Liver suspensions from G2, G3, G4 and G5 were prepared with 1 mL saline in a tissue homogenizer. Next, 0.1 mL of suspension was placed on a slide to identify tissue cysts under 10× magnification. The parasite load was then calculated for each group [42].

### 2.5. Molecular Identification

Liver tissue specimens (20–25 mg) from sacrificed animals were washed three times using sterile phosphate-buffered saline (PBS) (pH 7.4). Next, DNA was extracted from tissue samples from all groups using a GF-1 Tissue DNA extraction kit (Cat.-No.GF-TD-050, Vivantis Co., Malaysia) according to the manufacturer’s recommended protocol. Real-Time PCR (RT-PCR) was performed using ViPrime PLUS Taq qPCR Green Master Mix I (SYBR Green Dye, Cat QLMM12 Vivantis Co., Selangor Darul Ehsan, Malaysia). The PCR reaction and conditions (40 cycles) were based on a previously described protocol [47] using a set of primers designated with Laser gene DNA star software V15 (Table 1).

### 2.6. Histopathological Examination

Samples from liver and lung tissues were fixed in neutral buffered formalin (10%), dehydrated in an ethyl alcohol series, cleared with xylol, and then embedded in paraffin. Slices of 3–5 μm thickness were prepared and stained with hematoxylin and eosin (H&E) [48]. The stained sections were then examined for histopathological changes and the presence of parasite cysts within the tissue.

### 2.7. Histopathologic Scoring

Each animal was scored based on the tissue histopathological examination results [49]. Samples were scored quantitatively and semiquantitatively based on visual inspection of a minimum of 10 sections from each group. Photographs were taken at a magnification of 40×. Tissue alterations were scored according to set criteria: 0 = (absence of lesions), 1 = (1–10% extent of lesions), 2 = (11–40% extent of lesions), 3 = (41–50% extent of lesions), and 4 = (51–100% extent of lesions) [50]. Liver sections were analyzed for the number of parasites present and the number of inflammatory foci present according to a previously described method with minor modifications [51]. Lung tissue sections were scored according to the number of parasites, alterations in bronchioles, alveolar structure changes, and vascular changes. The nature and extent of the lesions and their frequency in the tissue were assessed [47,52]. All analyses were performed by two pathologists.

### 2.8. Statistical Analysis

Data (mean ± standard deviation) were compared between groups using a *t*-test performed with the Statistical Program for Social Science version 2. Histopathological scoring data were compared using one-way analysis of variance (ANOVA) with Tukey’s post hoc multiple comparison test with GraphPad Prism, version 5 (San Diego, California, CA, USA) [53]. A *p*-value < 0.05 was considered to indicate a statistically significant difference.

## 3. Results

### 3.1. Parasitic Load

**Figure 2:**
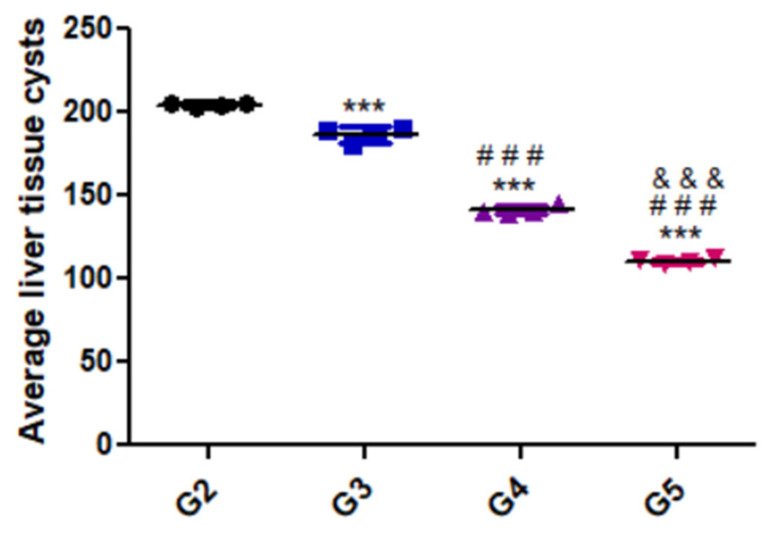
Average liver tissue cysts of the different experimental groups. Data are expressed as means ± standard deviations. Significant differences (G2 vs. other groups are marked by asterisks), (G3 vs. G4 and G5 are marked by #), ((G4 vs. G5 are marked by &) all through one-way ANOVA with Tukey’s post hoc test: ***, ###, &&& *p* ≤ 0.001).

Figure 2 presents the variation in parasite load in the liver tissues of the different groups. As shown, there was a substantial decrease in parasitic burden in the group treated with the combination of WGO and propolis (G5) compared with the positive control and the other treated groups.

### 3.2. Molecular Results

The DNA concentration of the *Toxoplasma P29* gene (GRA7) was quantified after treatment in all groups. As shown in Table 2, all samples gave positive results with clear variations in the product quantities. However, there was only a statistically significant difference between the untreated control group (G2) and the group treated with a combination of WGO and propolis (G5). A melting curve analysis was performed to rule out the presence of non-specific PCR.

### 3.3. Histopathological Findings

**Figure 3:**
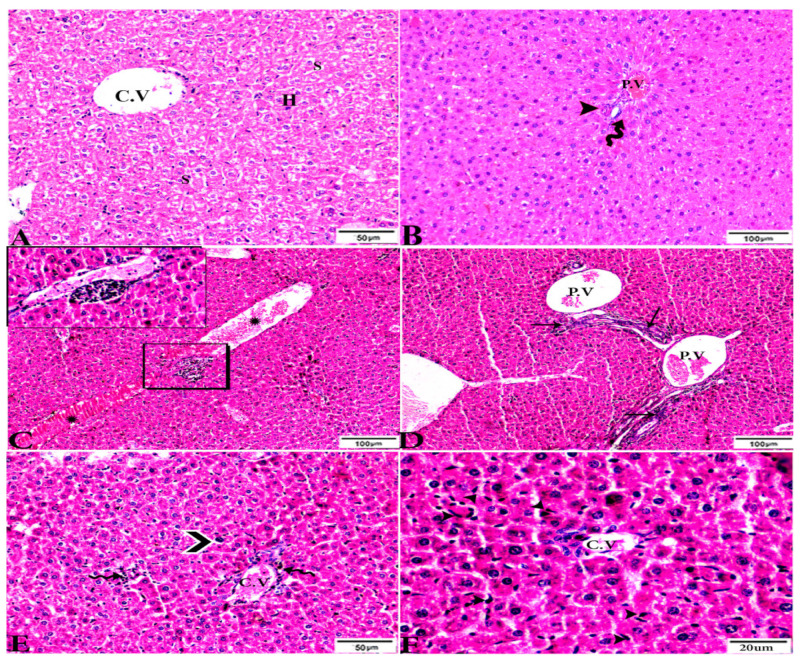
Photomicrograph of H&E-stained liver tissue sections from mice in the experimental groups: (**A**,**B**): Liver sections from the negative control group (G1) demonstrating: (**A**): normal hepatic architecture comprising a normal central vein (C.V), sinusoids (S), and hepatocytes (H); and (**B**): normal portal triad structures: hepatic artery (arrowhead), portal vein (P.V), and bile duct (zigzag arrow). (**C**–**F**): Liver tissue sections from mice in the *Toxoplasma gondii*-infected positive control group (G2) showing: (**C**): severe vascular congestion (stars), focal mononuclear cellular aggregations (selected squares), and coagulative necrosis in hepatocytes; (**D**): dilated and congested PV, longitudinal fibrous tracts connected between P.V (arrows); (**E**,**F**): congested C.V, multiple bradyzoites (zigzag arrows), tissue cyst (arrowhead), and marked Kupffer cell reactions (E, arrowheads). The scale bar is provided below each image.

**Figure 4:**
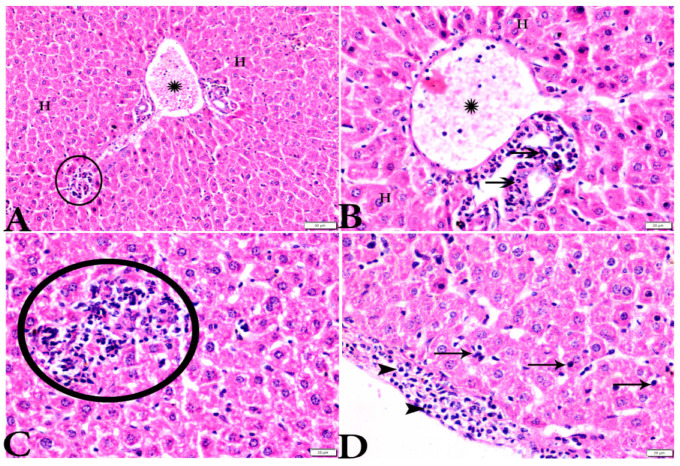
Photomicrograph of H&E-stained liver tissue sections from *Toxoplasma gondii*-infected animals treated with propolis (G3) showing: (**A**,**B**): severe vascular congestion in the portal vein (P.V) infiltrated with inflammatory cells (stars), more or less normal hepatocytes (H); and (**B**): aggregated bradyzoites (arrows) at portal area. (**A**,**C**): Focal area of mononuclear cellular infiltration (circles). (**D**): Multiple bradyzoites present in Gilson capsule (arrowheads) and bradyzoites in hepatocytes (arrows). The scale bar is provided below each image.

**Figure 5:**
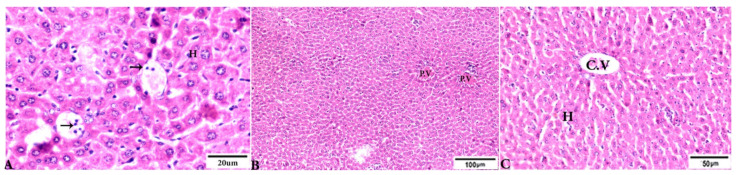
Photomicrograph of H&E-stained liver tissue sections from mice in the experimental groups: (**A**): liver tissue sections from mice in the *Toxoplasma gondii*-infected group treated with wheat germ oil (G4) showing normal hepatocellular structure (H); parasitic tissue cyst contains bradyzoites (arrows). (**B**,**C**): Liver tissue sections from mice in the combined propolis and wheat germ oil treatment group (G5) showing: (**B**): normal portal structure with normal portal vein (P.V), normal central vein (C.V), and normal hepatocytes (H). The scale bar is provided below each image.

**Figure 6:**
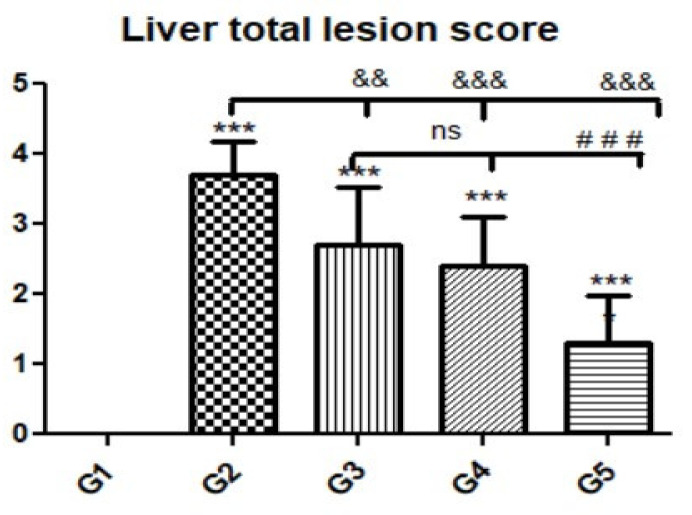
Quantitative and semiquantitative measurements of total lesion scores in liver tissue sections in the experimental groups. Data are expressed as the mean ± standard deviation. Significant differences (control group vs. other groups) are indicated by asterisks *, G2 vs. G3, G4, and G5 are marked by #, and G3 vs. G4 and G5 are marked by &. All tests were one-way ANOVA with Tukey’s post hoc test ns: non-significant && *p* ≤ 0.01, ***, ###, &&& *p* ≤ 0.001.

**Figure 7:**
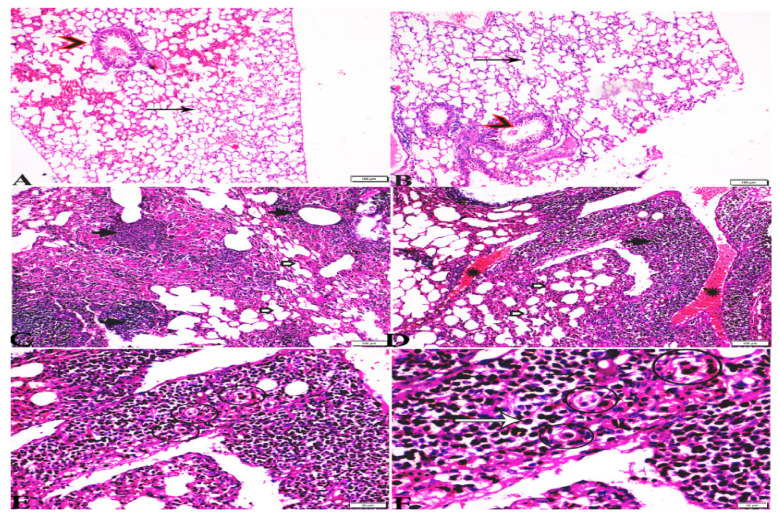
Photomicrograph of H&E-stained lung tissue sections from mice in the experimental groups: (**A**,**B**): lung tissue sections from the negative control group (G1) showing: normal bronchiolar structure (arrow heads), normal alveolar tissue (arrows). (**C**–**F**): Lung tissue sections from the *T. gondii*-infected positive group (G2): (**C**,**D**): collapsed alveoli (white arrows), multiple focal lymphoid hyperplasia (black arrows), and severe vascular congestion (stars). (**E**,**F**): Multiple tissue cysts containing bradyzoites (circles) and multiple diffuse bradyzoites (arrows). The scale bar is provided below each image.

**Figure 8:**
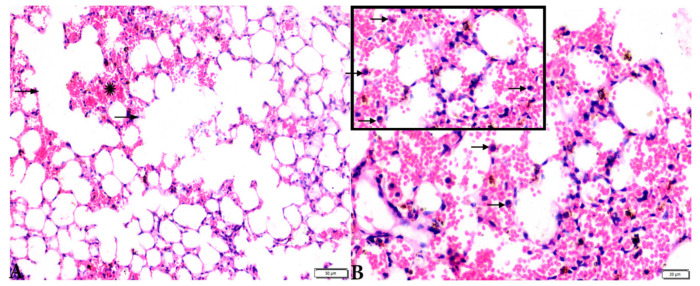
Photomicrograph of H&E-stained lung tissue sections from *T. gondii*-infected animals treated with propolis (G3) showing: (**A**): alveolar emphysema (arrows), interstitial hemorrhage (stars), and (**B**) infestation with multiple tissue cysts (arrows). The scale bar is provided below each image.

**Figure 9:**
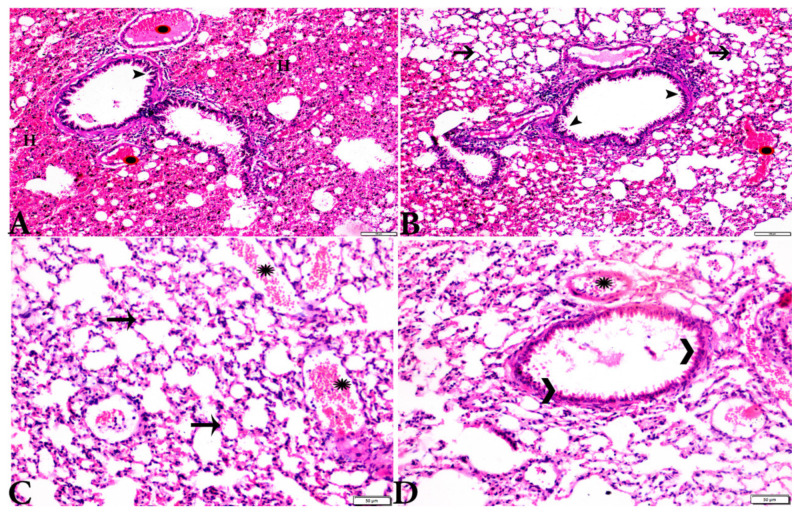
Photomicrograph of H&E-stained lung tissue sections from mice in the experimental groups: (**A**,**B**): lung tissue sections from *T. gondii*-infected mice treated with wheat germ oil (G4) showing: normal bronchiolar structure (arrowheads), congested blood vessel (stars), hemorrhage (H), and collapsed alveoli (arrows). (**C**,**D**): Lung tissue sections from the group treated with the combination of propolis and wheat germ oil (G5) showing: normal alveolar tissue (arrows), normal bronchiolar structure (arrowheads), and vascular congestion (stars). The scale bar is provided below each image.

To investigate the effects of propolis, WGO, or the combination of both substances on *T. gondii*-induced infection, liver (Figure 3, Figure 4, Figure 5 and Figure 6) and lung (Figure 7, Figure 8 and Figure 9) tissues from different groups were examined histopathologically. Liver sections (Figure 3A,B) from control noninfected mice (G1) demonstrated normal hepatic architecture, comprising a normal central vein and normal sinusoids between hepatic cellular cords. Normal portal triad structures (hepatic artery, portal vein, and bile duct) were also observed. In contrast, severe damage, multiple tissue cysts, congested vessels, obvious inflammation, coagulative necrotic foci, and numerous tissue cysts were observed in the liver (Figure 3C–F) of *T. gondii*-infected untreated mice (G2). Attenuated or moderate histological evidence (moderate inflammation and fewer necrosis foci) in hepatic tissues and a relatively high number of tachyzoites (Figure 4A–D) were observed in liver tissues of *T. gondii*-infected mice treated with propolis (G3). Treatment with WGO only (G4) ameliorated the histopathological changes (mild inflammation and normal hepatocellular structure), and a lower number of tachyzoites was observed (Figure 5A). Notably, liver tissue sections from *T. gondii*-infected mice treated with a combination of propolis and WGO appeared morphologically indistinguishable from uninfected controls (Figure 5B,C). Quantitative and semiquantitative analysis of the severity of inflammation, necrosis of liver sections, and the number of parasites present in tissue fields of different groups was performed (Figure 6). The results showed a significant increase in pathological lesions in *T. gondii*-infected untreated mice (G2) compared with the control group, whereas significantly reduced pathologic alterations were observed in the propolis (G3), WGO (G4), and combination treatment (G5) groups.

**Figure 10:**
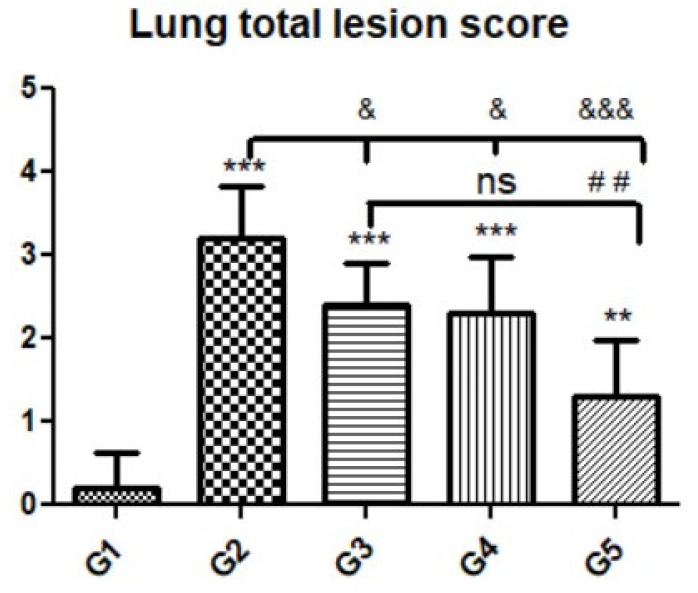
Quantitative and semiquantitative measurements of total lesion scores in lung tissue sections among the experimental groups. Data are expressed as the mean ± standard deviation. Significant differences (control group vs. other groups) are indicated by asterisks *, G2 vs. G3, G4, and G5 are marked by #, and G3 vs. G4 and G5 are marked by &. All tests were one-way ANOVA with Tukey’s post hoc test: ns: non-significant,**, ##, *p* ≤ 0.01, ***, &&& *p* ≤ 0.001.

Microscopic examination of lung tissues obtained from the negative control group (G1) (Figure 7A,B) revealed a normal bronchiolar structure and normal alveolar tissue. In contrast, lung tissue obtained from the positive control group (G2) showed severe damage, congested blood vessels, collapsed alveoli, multiple focal lymphoid hyperplasia, the presence of multiple tissue cysts containing bradyzoites, and multiple diffuse bradyzoites in the infected alveolar tissue (Figure 7C–F). Lung tissue sections obtained from infected animals treated with propolis (G3) exhibited moderate damage, alveolar emphysema, interstitial hemorrhage, infestation with multiple bradyzoites, and multiple tachyzoites (Figure 8A,B), while normal bronchiolar structure, mild damage, congested blood vessels, hemorrhage, and collapsed alveoli were observed in lung tissue specimens from animals treated with WGO (G4) (Figure 9A,B). Lung tissue from the G5 group showed normal histology and bronchiolar structure, normal alveolar tissue, and normal peribranchial lymphoid cellular structure (Figure 9C,D). Based on histopathology scoring (Figure 10), the severity of parasitic tissue cyst number, interstitial hemorrhage, blood vessel congestion, and bronchiolar epithelium structure were significantly improved (*p* ≤ 0.05) in all experimental treatment groups (G3, G4, and G5) compared with the positive control group (G2).

## 4. Discussion

Treatment of the chronic stage of toxoplasmosis remains challenging due to a lack of effective drugs; of the available drugs, many cause hematologic and renal toxicity and have poor diffusion [54]. Furthermore, many chemotherapeutics lead to severe side effects and are not well tolerated by humans and animals. Clinical failure and drug resistance are also issues. As effective, nontoxic novel drugs against chronic toxoplasmosis are still out of reach, many studies have tried to identify natural alternatives [55]. The current study explored the benefits of propolis, WGO, and a combination of both against chronic toxoplasmosis in mice as assessed by parasitic load and histopathological changes to the liver and lungs following treatment. In a previous study [56], we reported the ameliorative effects of WGO and propolis on the parasite burden in the uterus, brain, and kidney during chronic toxoplasmosis in mice. However, the potential influence of this combination on the parasite burden and histopathological changes in liver and lung tissues were not examined. The use of these drugs during the chronic stage of infection is essential to reduce cyst load in the host.

The present work found a substantial drop in parasitic burden in mice treated with propolis (G3) versus the untreated control group (G2). It should be stressed that several previous studies have reported the antiparasitic action of propolis against various intracellular and extracellular pathogenic protozoa, principally owing to its plant secondary metabolite content (phenolics and terpenoids), through various mechanisms of action, including induction of cell lysis, disruption of phospholipid metabolism, and reduction of essential lipids of pathogens such as phosphatidyl glycerol and phosphatidyl inositol (PI) [33,55]. Propolis has also been shown to have antiparasitic effects against protozoan parasites. The present findings are consistent with a previous study [57] reporting the obvious effect of propolis in reducing the number of cysts in rats experimentally infected with chronic toxoplasmosis. Furthermore, these results highlight the capability of propolis to restore the histological structure of the studied organs and suppress the multiplication of *T. gondii* tachyzoites, which is consistent with the findings of a prior study [57]. A previous in vitro report demonstrated the antitoxoplasma activity of an ethanolic extract of propolis [58]. Higher concentrations of propolis could directly increase the in vitro precipitating factor and trap heavier compounds that effectively destroy the tachyzoite stage of the parasite. Another study reported a significant increase in specific antibody titers (IgM and IgG) and decreased serum cytokine levels (IFN-γ, IL-1, and IL-6) in rats infected with *T. gondii* treated with propolis [59]. Propolis typically contains several phenolics, including rosmarinic acid and apigenin, that induce physical injury via cell lysis and cytoplasmic condensation [60]. Propolis also influences hydrogenosome metabolism, which is responsible for energy production in eukaryotes such as protozoa [61]. Other chemical compounds, including lupane, maslinic acid, ursolic acid, and limonene, identified in propolis are known to induce morphological deviations, promote apoptosis, and inhibit critical metabolic proteases and enzymes [37].

The current study is a pioneering work in exploring the antiparasitic activity of WGO against *T. gondii* in experimentally infected mice. As shown, there was a substantial decline in the liver parasite burden in mice treated with GWO (G4) compared to the untreated positive control group (G2) and the propolis-treated group (G3). The phytochemical mechanisms of wheat involve alkaloids, flavonoids, steroids, and saponins. Wheat kernels contain 2–4% germ, which is the richest natural source of tocopherol, B vitamins, and protein and, thus, of great biological value [62]. The antioxidant constituents of wheat germ extracts show antitrypanocidal effects, as represented by the ability to reduce parasitemia and the severity of disease [63]. Fermented wheat extract also has an antiproliferative action that targets nucleic acid synthesis enzymes and has analgesic, antimicrobial, anti-inflammatory, antioxidant and immunological effects [64]. According to the available literature, WGA exhibits a notable inhibitory effect against trophozoites of *Trichomonas vaginalis.* It also interferes with the function of surface glycoproteins involved in *Giardia* attachment [24]. Given the above-mentioned properties, WGO is a promising candidate for the treatment of chronic toxoplasmosis.

Molecular methods are considered effective for the identification and quantitation of parasitic loads in infected tissues [65,66]. The current study estimated the effects of herbal treatment on the growth and replication of *T. gondii* using real-time PCR through targeting detection and quantification of the *p29* (*GRA7*) gene. A previous study [47] revealed that this gene is present in the compact pellets of the parasite and secreted by bradyzoites and is thus likely valuable for detecting that specific phase of infection [67]. As shown in Figure 2, the untreated control group (G2) had the highest parasitic burden, while animals treated with the combination of propolis and WGO (G5) had the lowest parasite load, confirming the parasitological and histopathological findings.

The histological findings in the liver and lung tissue from infected untreated animals were in line with several previous studies. For example, liver tissue from *Toxoplasma*-infected mice showed disturbed architecture, extracellular tachyzoite collection, Kupffer cell hyperplasia, and diffuse hepatocyte ballooning with focal fatty degeneration, in addition to various other histopathological lesions [25]. A previous study [57] reported that hepatic tissues of animals infected with *Toxoplasma gondii* showed massive edema of the liver capsule. Furthermore, all portal tracts showed moderate to severe inflammatory infiltration with lymphocytes and plasma cells. Marked vascular dilatation and congestion were seen in all hepatic vessels, including central and portal veins as well as sinusoids. Infected humans and naturally occurring animal cases of pulmonary toxoplasmosis have also been documented [68,69]. In the present work, histopathological examination of liver and lung tissues of all studied groups aimed to verify the results of parasitological and molecular analyses. Our histological analysis of liver and lung tissues showed that treatment with propolis and WGO significantly reduced histopathological alterations and inflammation brought on by *T. gondii* infection. Interestingly, improvement was more significant in the combination group than in the groups treated with WGO or propolis alone.

## 5. Conclusions

Taken together, the findings of this study have demonstrated the promising effects of WGO and propolis against chronic toxoplasmosis in infected mice. Specifically, treatment restored histopathological changes in the liver and lungs. Further research is warranted to explore the immunological mechanisms and mechanistic pathways underlying these effects and the potential role of melatonin receptors in the antitoxoplasmal effects of WGO and propolis.

## Figures and Tables

**Table 1:** Primer set used for real-time polymerase chain reaction (RT-PCR) in the present study.

P29 Q-f	CAGCATGGATAAGGCATCTG
P29 Q-r	GTTGCTCCTCTGTTAGTTCC

**Table 2:** Mean CT value and concentration of the parasite in the liver tissues of experimental groups.

Groups	Sample Number	CT Value	Concentration (µg/µL)	Significance
G1 (noninfected, nontreated)	C-	No Ct	None	-
G2 (infected, nontreated)	1	23.22	0.0284	-
	2	19.11	0.0361	
	3	20.06	0.0069	
G3	4	25.28	0.1459	Nonsignificant
	5	26.55	0.0106	
	6	28.54	0.0553	
G4	7	29.25	0.1740	Nonsignificant
	8	32.13	0.1126	
	9	31.34	0.1330	
G5	10	33.37	0.1568	Significant (*p* < 0.05)
	11	36.13	0.1056	
	12	36.26	0.2080	

## Data Availability

Not applicable.
